# Mannheim’s peritonitis index in the prediction of postoperative outcome of peritonitis

**DOI:** 10.1590/0100-6991e-20222991_en

**Published:** 2022-08-25

**Authors:** LAÍS DOS SANTOS GUEIROS, CLÁUDIO MEDINA DA FONSECA, NATHALIA MARIA DIAS MORAES DUARTE, OLÍVIA SOUZA ANTUNES

**Affiliations:** 1 - Hospital Santa Casa de Misericórdia de Vitória, Cirurgia Geral - Vitória - ES - Brasil; 2 - EMESCAM, Medicina - Vitória - ES - Brasil

**Keywords:** Peritonitis, Abdomen, Acute, Sepsis, Mortality Registries, Peritonite, Sepse, Abdome Agudo, Mortalidade

## Abstract

**Objective:**

evaluate the effectiveness of MPI to predict mortality in patients with peritonitis in Santa Casa de Misericordia de Vitoria Hospital (HSCMV).

**Methods:**

a longitudinal observational cohort retrospectively study, with a sample of 75 patients diagnosed with peritonitis between January 2010 to December 2 of 2015, in HSCMV and with all the necessary criteria for the calculation of IPM.

**Results:**

we found a profile of the patients, 33 female and 42 male, mean age 42 years, 11 deaths and 14.67% mortality percentage. Comparing the MPI variables into two groups (survivors and deceased) was found that older than 50 years, presence of malignancy and patients with organ dysfunction have statistical significance for mortality, with p<0.05. The MPI ranged between 4-41 points, with average of 21.2 points. However, among the dead, the score ranged from 23 to 41, with a mean of 32.8. Therefore, the cutoff point of 27 points was established by evaluating the best value of Kappa concordance index, and through it were calculated: 90.90% sensitivity and specificity of 78.13% by the ROC curve.

**Conclusion:**

based on these results, it was established that the MPI was effective in estimating the risk of death when the index reaches values = 27 points. Categorizing patients into different risk groups helps in determining a better prognosis and defining operative risk, thus contributing to the choice of the surgical procedure nature.

## RESUMO

**Objetivo:**

avaliar a efetividade do índice de peritonite de Mannheim (IPM) para predizer mortalidade em pacientes com peritonite no Hospital Santa Casa de Misericórdia de Vitória (HSCMV).

**Método:**

Coorte longitudinal, observacional retrospectivo, com amostra de 75 pacientes diagnosticados com peritonite entre janeiro de 2010 a 02 de dezembro de 2015 no HSCMV, com todos os critérios necessários para o cálculo do IPM.

**Resultados:**

Encontrou-se um perfil dos pacientes, sendo 33 do sexo feminino e 42 do masculino, idade média de 42 anos, 11 óbitos e percentual de mortalidade de 14,67%. Comparando as variáveis do IPM em dois grupos (sobreviventes e falecidos), constatou-se que idade maior do que 50 anos, presença de malignidade e pacientes com disfunção de órgãos tiveram significância estatística para mortalidade, com p<0,05. O IPM variou entre 4 e 41 pontos, com média de 21,2 pontos. No entanto, entre os falecidos o escore variou de 23 a 41 pontos, com média de 32,8. Sendo assim, foi estabelecido o ponto de corte de 27 pontos através da avaliação do melhor valor do Índice Kappa de concordância, e através dele foram calculados: sensibilidade de 90,90% e especificidade de 78,13% através da curva ROC.

**Conclusão:**

Com base nesses resultados, foi visto que o IPM foi eficiente em estimar o risco de morte, sendo esse identificado quando o índice atinge valores = 27 pontos.

## INTRODUCTION

Peritonitis is defined as an inflammatory process of the peritoneum caused by any agents, such as bacteria, fungi, viruses, drugs, digestive secretions, granulomas, and foreign bodies. The clinical spectrum of peritonitis can also be classified according to the pathogenesis as primary, secondary, or tertiary peritonitis^1^.

Peritonitis is still one of the most important infectious problems that a surgeon must face. Despite progress in antimicrobial agents and intensive care treatment, the mortality rate from peritonitis exceeds 10 to 20%, which remains high^2^.

Therefore, scoring systems are necessary that allow the determination of the severity of intra-abdominal infection, to ratify the effectiveness of treatment, to assist in the calculation of individual risk for selection of patients who may need an earlier surgical approach, and to provide sufficient prognosis data.

The Mannheim Peritonitis Index (MPI) was developed by Wacha and Linder in 1983 for a retrospective study with 1,253 patients with peritonitis, which considered 20 possible risk factors. Of these, only eight proved to be of prognostic importance and were included in the MPI, classified according to their predictive power^3^ ^,^ ^4^. The MPI aimed to classify the severity of peritonitis or intra-abdominal infections, as well as to identify patients who need rapid intervention and earlier treatment, using parameters easily collected through clinical examinations and surgical exploration. The MPI considers age, sex, organ dysfunction, presence of malignancy, origin, evolution time >24h, extent of peritonitis, and characteristics of peritoneal exudate. Different values are assigned to each parameter, and the final score ranges from zero to 47 (Table 1). Patients with a score greater than 26 were defined as having a high mortality rate from severe peritonitis, with good specificity (79%), sensitivity (84%), and accuracy (81%)^3^ ^,^ ^4^.

**Table 1:** Mannheim Peritonitis Index.

Factor	Adverse	Points	Favorable	Points
Age	>50	5	<50	0
Sex	female	5	male	0
Organ Dysfunction	present	7	absent	0
Malignancy	present	4	absent	0
Evolution time	>24 hours	4	<24 hours	0
Origin	non-colonic	4	colonic	0
Extent of peritonitis	generalized	6	localized	0
Peritoneal exudate	fecaloid	12	clear	0
	purulent	6		
Organic dysfunction				
Kidney			Creatinine >177umol/L	
			Urea >167mmol/L	
			Oliguria <20mL/h	
Lung			PO^2^ <50 mmHg	
			PCO^2^ >50 mmHg	
Shock			Hypodynamic or Hyperdynamic	
Bowel obstruction			Paralysis >24 hours or complete mechanical obstruction	

Source: Correia, 2001.

We carried out this study to assess the effectiveness of MPI in the prognosis of patients with peritonitis in HSCMV, since there are few published studies to assess the validity of this prognostic index at the national (Brazilian) level. The aim is to categorize patients into different risk groups to determine the best prognosis and the operative risk, thus contributing to the choice of the operative procedure.

## METHODS

After approval by the EMESCAM Human Research Ethics Committee (CEP) under number 50831415.0.0000.5065, we performed a retrospective cohort study. We used descriptive statistical techniques for minimum sample calculation, considering significance of 0.05, test power of 80%, and a two-tailed test, which rendered a sample of 54 patients.

We started the selection of patients by the MV200 care system, with the codes of surgical procedures that fit the hospital profile and probable operative approaches to peritonitis. The selected procedures were exploratory laparotomy, appendectomy, laparoscopic appendectomy, surgical treatment of digestive tube diverticulum, and partial gastrectomy, with or without vagotomy. We included patients of both sexes, older than 15 years, admitted to the HSCMV from January 2010 to December 2, 2015. We excluded patients with incomplete data in their medical records necessary to calculate the MPI, as well as those who died within the first 24 hours of hospitalization.

The MV2000 online medical record system was implemented at the HSCMV in 2010, but it only allows for search medical records based on codes of procedures performed and not by diagnosis or ICD-10 code, which is a limiting factor for research.

Through the research of the aforementioned procedures, we pre-selected 1,285 patients. However, only 75 fit the diagnosis of peritonitis with all the inclusion criteria, contained in the collection form (Appendix): both sexes, older than 15 years, surgical description documented in medical records confirming diagnosis of peritonitis and with characteristic of the exudate found, non colonic sepsis origin, extension of peritonitis, presence or absence of malignancy, presence or absence of organ dysfunction (intestinal obstruction/paralysis ≥24h or complete mechanical obstruction, oliguria <20mL/h, creatinine >177umol/L or 2.32mg/dL, urea >67mmol/L or 467.8mg/dL, hypodynamic or hyperdynamic shock, PO^2^ <50mmHg, PCO^2^ >50mmHg), and evolution time >24h.

Only 32 patients met all criteria. Due to the small number of patients, it was necessary to include the 43 patients who did not have arterial blood gases performed at admission, but who were stable on physical examination and had other laboratory tests within the normal range, excluding possible organ dysfunction.

We stored the data in an Excel 2013 spreadsheet, which included the following data: patient care code, age, sex, urea, creatinine, oliguria, PO^2^, PCO^2^, presence or absence of shock, intestinal obstruction >24h, presence or absence of malignancy, evolution of the condition >24h, non colonic origin, extent of peritonitis (localized or diffuse), characteristics of the exudate (clear, purulent, or fecal), status (discharge or death), date of admission and hospital discharge/death, days of stay, and MPI score.

We used the Chi square or Fisher’s exact test (when at least one score is expected to be lower than five) to verify the association between qualitative variables. We used the Spearman’s correlation coefficient to verify correlation between quantitative variables. We performed comparison between groups using the nonparametric Mann-Whitney test. We used the ROC curve to calculate Specificity and Sensitivity.

The lowest possible MPI score is zero, when there are no risk factors, and the maximum is 47, when all risk factors are present. We divided the patients into two groups, based on the MPI cut-off point obtained, in which there was greater significance in predicting mortality by the profile of the HSCMV patients. We performed the statistical analysis with the SPSS software, version 23, with a significance level of 5%.

. 

## RESULTS

The 75 patients selected with a diagnosis of peritonitis ranged in age between 15 and 86 years, with a mean age of 42 and a standard deviation of 18.9. We observed that 42 patients were male, corresponding to 56%, and 33 were female, corresponding to 44%. Eleven patients died (14.67%).

**Figure 1:**
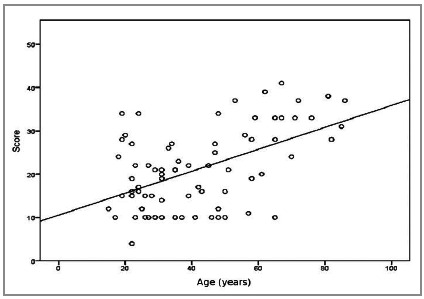
Dispersion of MPI according to age.

As shown in Figure 1, it is possible to identify a linear and positive relationship between age and the score, that is, for higher age values, there are higher scores, with a correlation coefficient of 0.418 and p-value=0.000.

As for causes, Acute Inflammatory Abdomen was the most prevalent, in 58 cases (77.3%). Among these, we found patients with acute appendicitis in grades III, IV, and V, with nine (12.0%), 44 (58.6%), and two cases (2.6%), respectively, in addition to one case of acute cholecystitis and one case of Pelvic Inflammatory Disease (PID), with 1.3% each. The second most prevalent cause was Perforated Acute Abdomen, with 14 cases (18.6%), one of perforated diverticulitis (1.3%), eight cases of postoperative peritonitis (10.6%), one case of ruptured bladder (1.3%), one case of gunshot wound (1.3%), and three cases of perforated ulcer (4.0%). The other causes are shown in Tables 2 and 3. The main factor associated with death was postoperative peritonitis (five deaths), corresponding to 45.5% of the total number of deaths.

**Table 2:** Distribution of the peritonitis causes.

Cause	Frequency	%
Acute Inflammatory Abdomen	58	77.33
Acute Hemorrhagic Abdomen	1	1.33
Acute Obstructive Abdomen	1	1.33
Perforated Acute Abdomen	14	18.67
Post-puncture Contamination	1	1.33
Total	75	100%

Source: authors.

**Table 3:** Distribution of specific peritonitis causes.

Cause	Frequency	%
Acute Inflammatory Abdomen		
Grade III appendicitis	9	12.00
Grade IV appendicitis	44	58.67
Grade V appendicitis	2	2.67
Cause	Frequency	%
Acute cholecystitis	2	2.67
PID	1	1.33
Acute Hemorrhagic Abdomen		
Ruptured ovarian cyst	1	1.33
Obstructive Acute Abdomen		
neoplasm	1	1.33
Perforated Acute Abdomen		
Perforated diverticulitis	1	1.33
Postoperative	8	10.67
Bladder rupture	1	1.33
Perforated ulcer	3	4.00
Trauma	1	1.33
Post-puncture Contamination		
Peritoneal dialysis	1	1.33
Total	75	100.00

Source: authors.

The preoperative duration was longer than 24 hours in 61 cases (81.3%). Purulent exudate was the most frequent, corresponding to 58 cases (77.3%). The extent of peritonitis was diffuse in 48 cases (64.0%). In only nine cases (12%), the peritonitis was of non colonic origin. Regarding organ dysfunction, there were 31 cases (41.3%) and only seven (9.3%) occurred in cancer patients, as shown in Table 4.

**Table 4:** Distribuição das variáveis do IPM entre pacientes que faleceram e sobreviveram.

Risk factor	Total (n=75)	Total (%)	Discharge (%)	Death (%)	p
Age >50 years	22	29.33	59.1	40.9	0.000
Female	33	44	81.8	18.2	0.520
Organ Dysfunction	31	41.33	35.5	64.5	0.000
Malignancy	7	9.30	14.3	85.7	0.000
Duration >24 hours	61	81.30	83.6	16.4	0.678
Non-colonic origin	9	12.00	66.7	33.3	0.121
Diffuse peritonitis	48	64.00	79.2	20.8	0.085
Risk factor	Total (n=75)	Total (%)	Discharge (%)	Death (%)	p
Exudate					
Clear	1	1.30	100	0	
Purulent	58	77.30	84.5	15.5	0.876
Fecaloid	16	21.30	87.5	12.5	

Source: authors.

When comparing the MPI variables in the two groups (survivors and deceased), we found that age greater than 50 years, presence of malignancy, and patients with organ dysfunction had statistical significance, with p<0.05.

**Figure 2:**
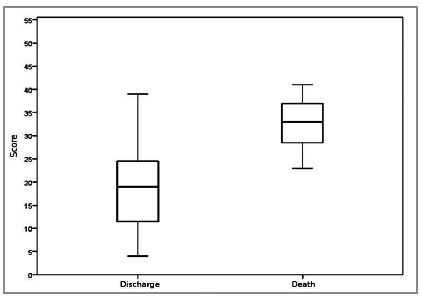
Median MPI according to outcome (discharge or death) using the nonparametric Mann-Whitney test, which showed statistical significance (p=0.000).

The MPI ranged from 4 to 41 points, with a mean of 21.2 points and a median of 21. However, among the 11 (14.7%) patients who died, the score ranged from 23 to 41, with a mean of 32.8 and a median of 33. Among the 64 (85.3%) patients who survived, the MPI ranged from 4 to 39, with a mean of 19.2 and median of 19 (Table 5 and Figure 2). 

**Table 5:** Variation of MPI according to outcome (discharge or death).

	Escore					
	Minimum	Maximum	Mean	Median	Standard deviation	Total (n= 75)
Discharge	4.0	39.0	19.2	19.0	8.2	64
Death	23.0	41.0	32.8	33.0	5.6	11

Source: authors..

**Figure 3:**
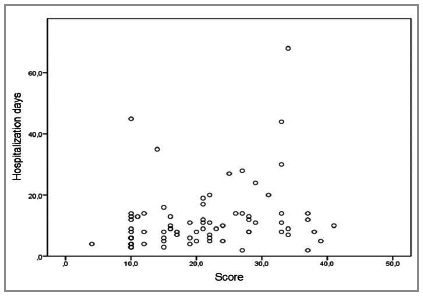
Dispersion of hospitalization days according to MPI using Spearman’s non-parametric correlation, which showed statistical significance (p=0.015).

We observed an average length of hospital stay of approximately 12 days, with a minimum length of stay of 2 days and a maximum of 68. There was a positive and weak correlation, with a coefficient of 0.281 and a p value of 0.015, indicating a significant, but not linear, correlation (Figure 3).

**Figure 4:**
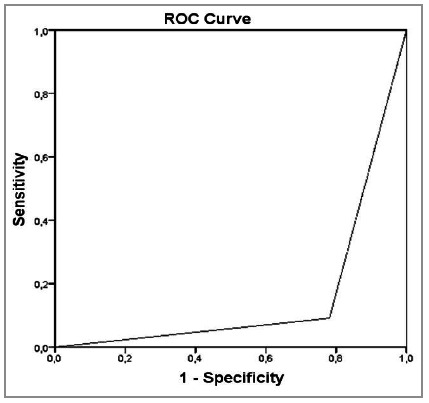
MPI Sensitivity and Specificity.

The outcome was associated with the score (<27 and ≥27) on a regular basis, the value of the Kappa index being 0.464, significantly greater than zero (p=0.000). The cut-off point of 27 points was obtained by evaluating the Kappa Index. Using this best cut-off point, we calculated a sensitivity of 90.9% and a specificity of 78.1% by the ROC curve (Figure 4). The MPI’s Positive Predictive Value was 71.4%, that is, the probability of an individual with a score ≥27 dying. The Negative Predictive Value was 98.0%, that is, the probability of an individual with a score <27 being discharged

**Figure 5:**
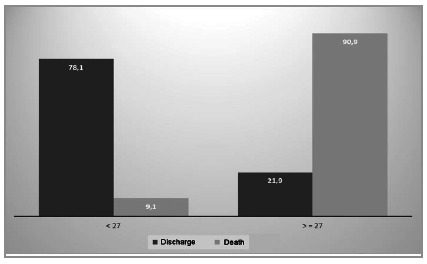
Distribution of MPI Scores according to outcome (discharge or death).

The mortality for patients with a score below 27 was 9.1%, and for the ones with a score ≥27, 90.9% (Table 6 and Figure 5). 

**Table 6:** Distribution of MPI Scores according to outcome (discharge or death).

		Outcome		Total
Score		Discharge	Death	
<27	n	50	1	51
	%	78.1%	9.1%	68.0%
≥27	n	14	10	24
	%	21.9%	90.9%	32.0%
	n	64	11	75
Total	%	100.0%	100.0%	100.0%

Source: authors.

## DISCUSSION

Early classification of the peritonitis severity can help to decide surgical and medical management. Therefore, scoring systems, such as the Mannheim Peritonitis Index, are needed to assist in risk stratification and assessment of new diagnostic modalities and therapeutic advances, as well as to compare treatment outcomes from different clinics.

By evaluating all risk factors among patients with peritonitis in HSCMV from 2010 to 2015, we were able to trace a profile that indicates higher mortality. The main causes of peritonitis were of non colonic origin. We noted that the most frequent specific diagnosis in HSCMV patients was acute appendicitis, but it was not the main factor of death, this being postoperative peritonitis. Of the risk factors evaluated, age greater than 50 years, organ dysfunction, and the presence of malignancy showed statistically significant associations with mortality. Regarding length of stay, there was a positive and non-linear correlation with patients’ scores, which indicates that it is a potential risk factor, but there is no relationship directly proportional to the score.

Looking at age, we observed that the patients who died were 64 years old on average, which agrees with the mean age of 60 years in other studies^5^ ^,^ ^6^.

For patients who survived, the MPI ranged from 4 to 39 points, with a mean of 19.2, which is higher than the mean of previous works^2^ ^,^ ^5^ ^,^ ^6^. However, it was equivalent to another reference^7^.

When comparing this work with others^2^ ^,^ ^5^ ^-^ ^7^, we observed similarity in the MPI values of the patients who died. We found values ranging from 23 to 41 points, with an average of 32.8. We observed a mortality rate of 14.6%, similar to the rate of 11.70% found in another article^5^.

Age greater than 50 years, presence of malignancy, and organ dysfunction were statistically significant, all with p=0.000. Of the patients who died, 81.8% were aged >50 years, with a mortality rate of 40.9%. Thus, mortality was proportional to the increase in age.

Regarding malignancy and mortality, we also found statistical significance. Of the 11 deaths in this study, 54.5% were cancer patients. This relationship had already been found^2^ ^,^ ^6^.

The presence of organ dysfunction increased the risk of death in patients with peritonitis (p=0.000), since in our study all patients who died had organ dysfunction. This result is similar to what was found in the literature^2^ ^,^ ^6^ ^,^ ^8^.

Mortality was not affected by sex and duration of peritonitis longer than 24 hours. All patients who died had disease duration longer than 24 hours, the same finding of other studies^2^ ^,^ ^6^.

The risk of death in patients with peritonitis of non colonic origin is higher than in those with a colonic origin, as found in another reference^2^. Non colonic origin is a risk factor for MPI. However, in the present study, this relationship was not proven, as in other previous works^6^ ^,^ ^8^.

We observed diffuse peritonitis in 48 patients, of whom 10 died and 38 were discharged. Of all 11 deaths, 90.9% had diffuse peritonitis. However, there was no significant difference between extension and mortality, as in another study^2^. Other studies6,8 have shown a significant relationship, though.

In this study, 77.3% of the patients had purulent exudate, and this type of exudate was found in 81.8% of those who died. This profile was also seen in other studies^2^ ^,^ ^8^.

The mean length of hospital stay for patients who were discharged was approximately 11 days, and for those who died, 14 days, which disagrees with a study that analyzed only cases of patients in intensive care^7^. In that, the total average value was approximately 12 days. There was statistical significance between days of hospitalization and score, in accordance with other series^7^. After analyzing Figure 3, we noted that this association was not linear, but positive. Probably, the higher the score, the greater the patient severity, with a greater probability of dying, of staying hospitalized for fewer days or, even, of being hospitalized for more days due to complications.

After analyzing the data for each factor of the score, we found that of the patients who died, 90.9% had a MPI greater than or equal to 27. Of those who did not die, only 21.9% had this score. Therefore, we can infer that the cutoff point for predicting mortality with MPI, based on the profile of patients treated at the HSCMV in the period from 2010 to 2015, is 27 points. The sensitivity for mortality is 90.9% and the specificity is 78.1%, similar to what was found in another study^2^, which showed sensitivity of 95.9% and specificity of 80.0%. In a literature reference^9^, there was sensitivity of 86.0%, specificity of 74.0%, and accuracy of 76.0%. In a study carried out at the Brazilian National Cancer Institute (INCA)^10^, the cut-off point found was 21 among cancer patients, whose mortality risk is higher; the sensitivity was 87.3% and the specificity, 41.2%.

The cut-off of 27 points presented in this study showed statistical significance. Therefore, we recommended using it to assess the prognosis in HSCMV patients. Thus, we created a standard care form for patients with suspected peritonitis to be used in the initial care in the emergency room of the HSCMV (Appendix C).

Some limitations of this study include the MV200 care system, which limited the search only for surgical procedure codes and not for ICD-10 or diagnoses, thus possibly enabling a selection bias. In addition, many data points needed to complete the MPI were not described in the medical records, thus, many patients were excluded from the study, reducing the sample.

The use of the MPI is recommended in the initial care of the patient to institute an early approach when a score range ≥27 points is reached. This stratification helps in determining the prognosis and defining the operative risk, thus contributing to the choice and planning of the operation, such as damage control or definitive procedure.

## CONCLUSION

Most patients who died had MPI ≥27 points, which indicates that MPI is efficient in estimating the risk of death and categorizing patients into risk groups.

We recommend that the MPI cut-off point should be adjusted for each hospital. Therefore, our current results can only be applied to hospitals with characteristics similar to the one of this study.

## References

[1] Wittmann DH, Schein M, Condon RE (1996). Management of secondary peritonitis. Ann Surg.

[2] Melgarejo EB, Castro MR, Luque GB, Trujillo NN (2010). Valor Predictivo de Mortalidad del Indice de Peritonitis de Mannheim. Rev Gastroenterol Peru.

[3] Muralidhar VA, Madhu CP, Sudhir S, Madhu S (2014). Efficacy of Mannheim Peritonitis Index (MPI) Score in Patients with Secondary Peritonitis. J Clin Diagn Res.

[4] Basnet RB, Sharma VK (2010). Evaluation of predictive power of Mannheim Peritonitis Index. Postgrad Med J.

[5] Teleanu G, Iordache F, Beuran M (2012). The Predictive Value of Mannheim Score in Patients with colon related peritonitis. Acta Medica Marisiensis.

[6] Bracho-Riquelme RL, Melero-Vela A, Torres-Ramírez A (2002). Mannheim Peritonitis Index Validation study at the Hospital General de Durango (Mexico). Cir Ciruj.

[7] Rodriguez H, García RP, Morales MP, Brambila VR, García VC (1999). Factores pronósticos asociados a mortalidad en pacientes com sepsis intrabdominal tratados em la unidad de terapia intensiva. Cir. & cir.

[8] Correia MM, Thuler LCS, Vidal EM, Schanaider A (2001). Prediction of death using the Mannheim Peritonitis Index in oncologic patients. Rev Bras de Cancerol.

[9] Biling A, Frolich D, Schildberg F (1994). Prediction of outcome using the Mannheim peritonitis index in 2003 patients. Br J Surg.

[10] Burger JA, Schöffel U, Sach M, Jacobs E, Kownatzki E, Von Specht BU (1995). Effects of peritonitis exudates on chemotaxis and phagocytosis of human neutrophils. Eur J Surg.

